# Transactions of the Medical and Physical Society of Bombay, Vol. I

**Published:** 1839-01-01

**Authors:** 


### Transactions of the Medical and Physical Society of Bombay. Vol. 1. 1838. Richardson, Cornhill.

Bombay has followed the example—longo intervallo—of its elder sister, Calcutta, in the publication of Transactions. What is Madras about? Are our professional brethren of the second Presidency of India torpid behind their tatties, and unable to rouse themselves to energy, like those of the eastern and western capitals? The volume which lies before us is respectable, and augurs well for its successors. Although it falls short of 400 pages, its contents are various and miscellaneous. The first article occupies 79 pages, and delineates the medical topography of Guzerat. It is from the pen of Mr. Gibson, and does him credit. Though valuable and interesting to the Bombay Establishment, it would not be sufficiently so to our readers in this far western longitude and gloomy climate. We must therefore pass it and many other papers over, in order to select such as are generally interesting to the profession at home.

The third paper makes us acquainted with another new disease of Indian growth, which bids fair to eclipse the famous cholera of 1817. It scourged various parts of our Asiatic possessions between the years 1815 and 1820, and appears to bear no small affinity to the plague of the Levant. On its first visitation it was generally ushered in by febrile symptoms, with slight cough, pain in the chest, and hæmorrhage. The same was observed in the famous plague of Athens. The author of the first paper on the subject gives us no detailed description of the disease except saying that, besides buboes—"all the other symptoms which have been enumerated as frequent in plague, were found in the different cases of this disease." It prevailed in Kallywar, Rutch, and part of Guzerat, causing great mortality.

Passing over 240 pages of the volume, we come to a paper by Mr. Hunter, entitled—

---

"CASES OF CARDIAC DISEASE AND OF TUBERCULAR PHTHISIS, OCCURRING IN THE 2D OR QUEEN'S ROYAL REGIMENT."

Ever since Mr. H. joined the Regiment in 1831, at Colaba, he has been struck with the frequency of cardiac and aortic diseases. He first thought it was, in some way, connected with purpura, which is very common at that station, but found afterwards that it prevailed at Poona, where purpura did not appear. It was frequently traced to attacks of rheumatism.

"But whether, or not, rheumatism be the first link in the morbid chain, a more efficient cause for hastening its progress, I am convinced, is the active duty a soldier undergoes whilst buttoned up in his accoutrements. These, by compressing the neck and chest, obstruct the circulation to such a degree, as to excite the heart to inordinate action, and consequent hypertrophy in the strong and muscular, or to dilatation in the weak and sickly; and in either case, particularly if there is any original disproportion of the organ, according to Laennec, a very frequent occurrence.

Again, in the former, the natural resiliency of the aorta being overcome by the inordinate force of the circulation, that vessel yields, dilates, and finally gives way, giving rise to aneurism.

It seems extraordinary that, now the effects of tight-lacing on females are so well known, a soldier, intended for the most active and long continued exertion, should be placed in a similar predicament, when that very exertion is required. Is it possible he could be placed under more unfavourable circumstances?

It is said it makes him look smart, so says the school-mistress; and no doubt it does to those who have been accustomed to associate perfection with such dresses and forms, but does it to the student of nature? Quite the reverse; to him it can be productive only of pain. It is true the form having in a great measure, acquired its natural set, before the recruit enlists, his chest is not so squeezed into a triangle, nor his waist unto that of a 'spaniora;' nevertheless, taking his active duty into account, the compression can scarcely be less detrimental, and more especially in that climate, from the perspiration it at the same time creates." 240.

The author after this exposé of Indian dandyism, relates some cases of the disease in question, some few particulars of which we shall state.

*Case* 1. A young man, ten years in India, had had rheumatic fever in England severely, but did not complain in India till two months before the date of report, when he began to experience dyspnœa on exertion or when swaddled in his accoutrements. This increased greatly, became complicated with cough, expectoration, but no fever. Pulse 80, firm and strong—pain in the left side of the chest. Was bled, with some relief. The physical signs are minutely detailed, but need not be stated here. The bruit de scie and bruit de soufflet, with violent impulse, and throbbing of the carotids, &c. indicated clearly enough the condition of the heart. In about a month there was aggravation of cough, with bloody expectoration, and utter inability to lie down. He soon afterwards died.

On dissection there was found serous infiltration into the cavities and cellular membrane—liver indurated and granular. Lungs in the different stages of inflammation, with purulent infiltration—heart enormously enlarged, with six or eight ounces of water in the pericardium. The parietes were thickened and there was disease both of the mitral and aortic valves.

By the way, our author diagnosed that there was no valvular disease—a prophecy which we should be very sorry to make, where there were the two bruits before alluded to. Mr. Hunter will be a little more cautious in future. For although we do not say that the bruits are *certain* signs of valvular disease, we apprehend that they are *generally* so. Two other and not very dissimilar cases are related; but present nothing of much interest. The same may be said of the cases of tubercular phthisis, which would seem to be detailed chiefly with the view of showing that Mr. Hunter is an expert auscultator. Of his talent in this way we have no doubt, and right glad are we to see the "inutile lignum," as the stethoscope was first called, so usefully employed in our Indian possessions.

With the two following very curious cases, taken from the Appendix, we must conclude our notice of this volume.

### Intestinal Sloughing.

*Case* 1. By Dr. Brown.—"This case occurred in Farrier C. McDonough, 3d Troop Horse Artillery, who was discharged from hospital, cured of fever, on the 17th November 1834. He returned to his duty, but was re-admitted into hospital on the 30th November: stated that for a day or two previously he had experienced frequent feverish feelings, a dull pain, with sense of twisting in the region of the abdomen, and attended by constipation.

On admission he had almost constant calls to stool. The evacuations were scanty, very offensive, and passed with much straining. The abdomen was slightly tumid; there was general tenderness, on pressure more felt however, on the left side: his countenance was extremely anxious and pallid: tongue slightly furred at the root, and centre, with raw and reddish-edges: frequent irritability of stomach: pulse soft, and rather quick: surface nearly natural, dejections thin, of a reddish colour, and extremely offensive.

With more or less of these symptoms he continued till 20th December, when, while straining at stool, there came away *per anum*, a tubular, membranous substance of about 25 inches in length: on examination this proved to be a portion of large intestine, which retained its natural calibre, but the walls of which were much thickened: on dissection the three coats could readily be separated: the fatty appendices, and the three longitudinal fibrous bands, were distinctly visible.

After the separation of this portion of intestine, the evacuations, though still dysenteric, were passed without straining, and often involuntarily; gradually, however, they became less frequent, and the power of retention returned. The nausea and retching ceased, but the least increase of diet never failed to be prejudicial. Bread and milk was that which suited best, and its use was continued till January 18th, when the appetite having become very keen, and the patient importunate for a change, chicken was allowed; much irritation resulted, and, though the former diet was resorted to, the patient continued very restless and uneasy, but without any other symptoms, till within about an hour of his death on the 20th January, when excruciating pain of the lower part of the abdomen was complained of.

*Inspection.* It is much to be regretted that Dr. Brown has lost the minute notes which he took of the appearances on dissection. The following statement is made from notes supplied by Mr. Bowstead, from recollection of the dissection, at which he assisted: their accuracy is confirmed by Dr. Brown.

The small intestines were so knotted together by adhesions that they could not be traced. The large intestines were much shortened, and not a vestige of the sigmoid flexure existed. There were morbid adhesions at the posterior part of the bladder, the lower portion of the rectum was much increased in capacity, and seemed to have been formed into a place of lodgment, where, in all probability, the intussuscepted bowel had for some time rested." 337.

The second case is still more curious than the first, since recovery took place.

*Case* 2. By Dr. Inglis.—" J. T. ætat. 40, admitted into the European Hospital, April 22d 1835, stated that for six or seven days he had been suffering from bowel-complaint with frequent and often ineffectual calls to evacuate the bowels. There was tenderness across the abdomen on pressure, with slight heat of skin, and frequency of pulse. On the 23d the tenesmus continued, and towards evening an increase of pain of abdomen was relieved by an anodyne enema and warm bath; on the 24th it is reported, that during the night there had been six copious, feculent evacuations without tenesmus, and during the day four evacuations scanty and mucous; on the 25th, five evacuations feculent and bilious; and 'at 8 P.M. there was expelled *per anum* a portion of intestine about seven inches in length. Says he was not sensible of its presence in the rectum, but first perceived it when partially expelled and retained at one end by the sphincter muscle; the gut appears to be in a putrid state.'

The patient continued in hospital with relaxed and irregular bowels till the 9th May, when he left much improved in health. He was the carpenter of a ship, and had made twelve voyages to India. Health in general good, and with the exception of one attack of cholera, had never suffered from serious disease: there was tendency to constipation, but to no considerable extent, and there never had been suffering from any other affection of the bowels." 346.

There may reasonably be entertained some doubts as to the fact of the above being actually a piece of intestine, since tubes of more than seven inches in length are sometimes discharged resembling intestine, but turning out to be only a secretion.

As we said before there very are many articles in this volume that will greatly interest the Indian practitioner, but which we cannot transplant to the pages of a European Journal. One, for example, is a very excellent account of the climate of *Mahabuleshwur* Hills, by J. Murray, Esq. These hills form part of the great Western Ghats, and a sanitarium or convalescent station has been fixed there, in about 18° of north latitude. It is open to the sea-breeze, and sheltered against the Easterly winds. It is situated at an elevation of 4500 feet. The climate is supposed to resemble very much that of the Cape of Good Hope. From recent information, however, we learn that Australia is becoming the favourite *sanitarium* for Indian invalids. When steam conveyance is established, and even without that, the voyage to Australia, and the fine climate of that strange land of Kangaroos, will, in a considerable degree, supersede the long and expensive voyage to Europe.

